# Entomopathogenic nematodes and their symbiotic bacteria: from genes to field uses

**DOI:** 10.3389/finsc.2023.1195254

**Published:** 2023-08-29

**Authors:** Eustachio Tarasco, Elena Fanelli, Carlo Salvemini, Yara El-Khoury, Alberto Troccoli, Alessio Vovlas, Francesca De Luca

**Affiliations:** ^1^ Department of Soil, Plant and Food Sciences, University of Bari “Aldo Moro”, Bari, Italy; ^2^ Institute for Sustainable Plant Protection (IPSP), Consiglio Nazionale delle Ricerche (CNR), Bari, Italy

**Keywords:** Steinernematidae, Heterorhabditidae, microbial control, survey, EPN native strains

## Abstract

The term “microbial control” has been used to describe the use of microbial pathogens (bacteria, viruses, or fungi) or entomopathogenic nematodes (EPNs) to control various insect pest populations. EPNs are among the best biocontrol agents, and major developments in their use have occurred in recent decades, with many surveys having been conducted all over the world to identify EPNs that may have potential in the management of insect pests. For nematodes, the term “entomopathogenic” means “causing disease to insects” and is mainly used in reference to the bacterial symbionts of *Steinernema* and *Heterorhabditis* (*Xenorhabdus* and *Photorhabdus*, respectively), which cause EPN infectivity. A compendium of our multiannual experiences on EPN surveys and on their collection, identification, characterization, and use in agro-forestry ecosystems is presented here to testify and demonstrate once again that biological control with EPNs is possible and offers many advantages over chemicals, such as end-user safety, minimal damage to natural enemies, and lack of environmental pollution, which are essential conditions for an advanced IPM strategy.

## Introduction

**Figure 1:**
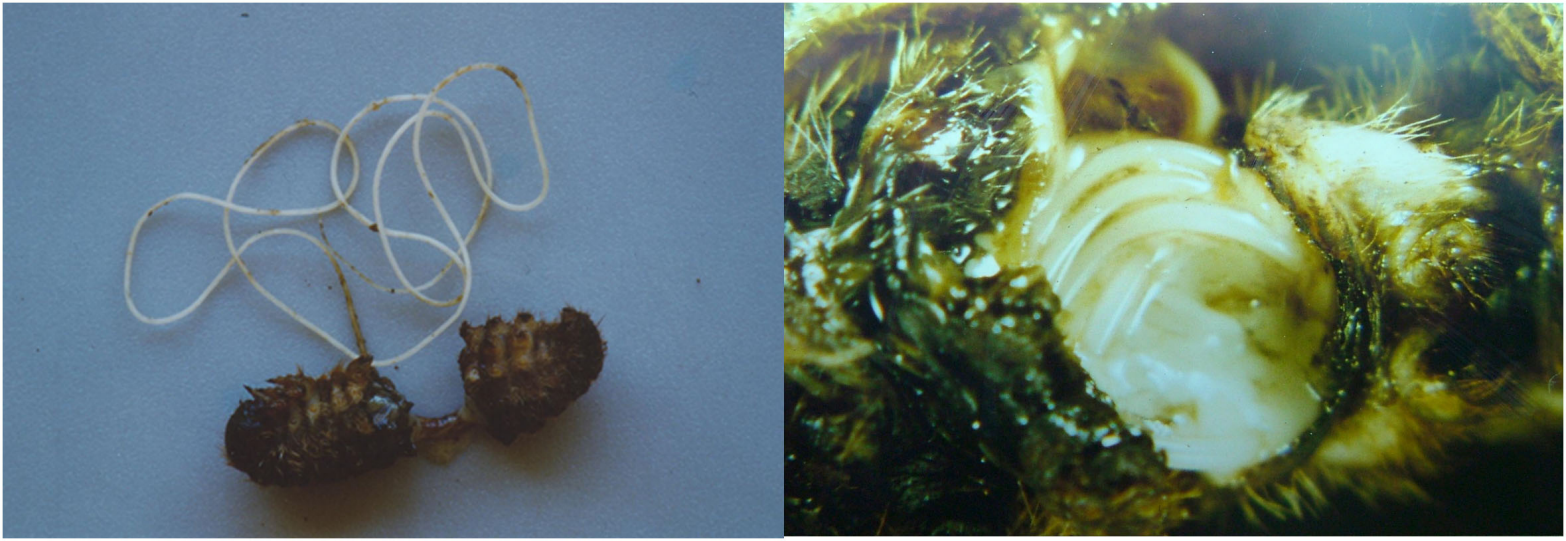
*Hexamermis* sp. (Nematoda, Mermithidae), entomoparasitic nematode predating on *Thaumetopoea pityocampa* larva.

The majority of nematodes are free-living organisms found in soil or in water. One-quarter of all nematodes are parasites of plants or animals (1), and, among the latter, some species are associated in various ways with insects. These relationships range from phoresy to symbiosis, and from commensalism to facultative or obligate parasitism (2, 3), and may involve insect parasitic nematodes (e.g., the natural parasite *Hexamermis* sp.) (**Figure 1**) or entomopathogenic nematodes (EPNs). They are frequently found in nature, infesting their hosts on the exoskeleton, or in the hemocoel, or nested in the reproductive, respiratory, digestive, or excretory systems. They can induce sterility; reduce fecundity, longevity, and the host’s ability to move; induce developmental delay; cause morphological changes; and ultimately host death may occur (4). Among more than 30 families of nematodes associated with insects, the families Steinernematidae Filipjev, 1934, and Heterorhabditidae Poinar, 1976 (order Rhabditida), are the ones that arouse the most interest. From a practical point of view in terms of hexapod control, nematodes within these two families are more accurately called entomopathogens because they exert their action in association with symbiotic bacteria (5–7).

## Biology and ethology of Steinernematidae and Heterorhabditidae

**Figure 2:**
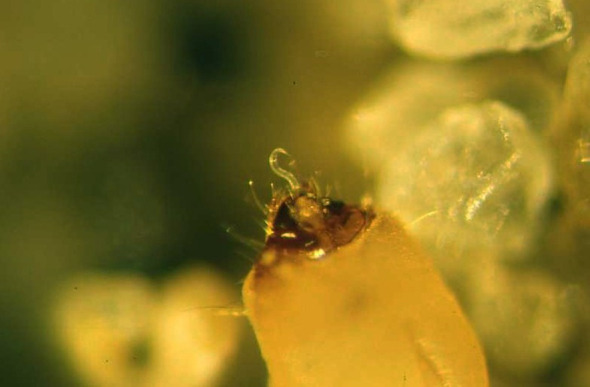
Infective juvenile (IJ) stage of *Steinernema feltiae* penetrating through the mouth of a young *Capnodis tenebrionis* larva.

**Figure 3:**
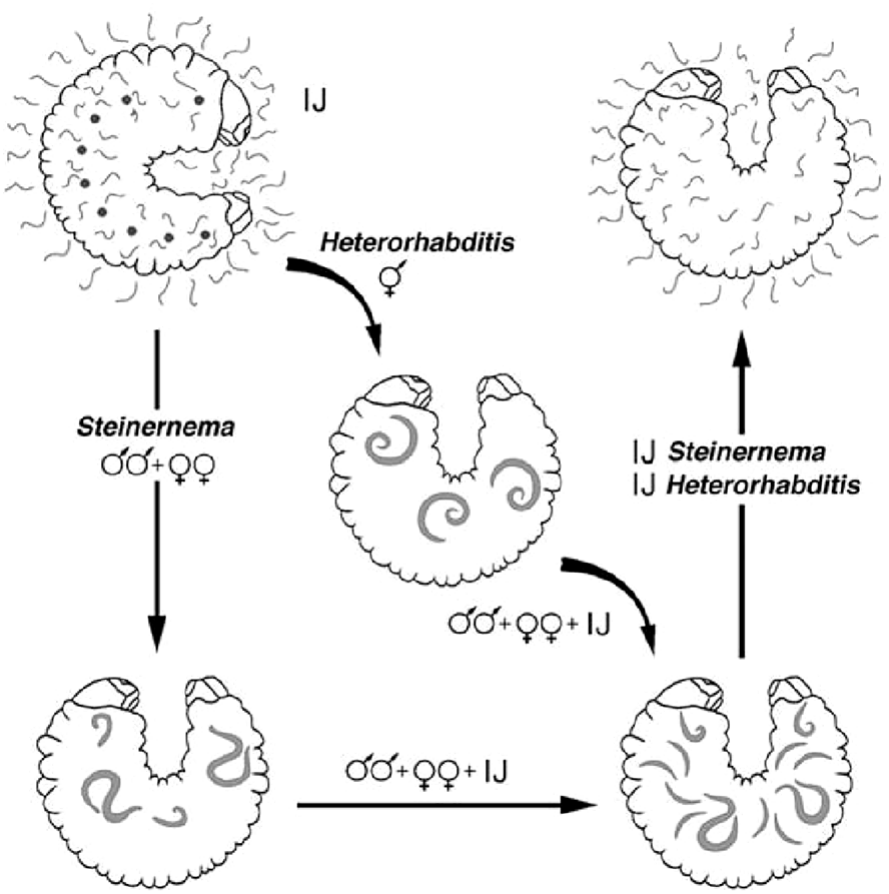
Biological cycle of *Steinernema* and *Heterorhabditis*.

The biological cycle begins with young females laying eggs in the substrate, but when they become older or hermaphrodite, the eggs hatch in the mother’s uterus (*endotokia matricida*) (8). In both Steinernematidae and Heterorhabditidae, it is the third stage, also known as the infective juvenile (IJ) stage, that initiates the infection. After reproduction and multiplication, the IJs abandon the cadaver, retaining the cuticle of the previous stage that protects them from dehydration, attacks from pathogenic fungi, and other forms of stress (9). Their subsequent movement through the soil in search of new hosts leads to the loss of this protective cuticle. Having identified the target host, IJs penetrate its body, preferably through natural openings (i.e., the mouth, anus, or stigmas) (**Figure 2**), after drilling its tracheae or intestines (10). *Heterorhabditis* spp., having a tooth in the anterior part of the body, have a comparative advantage because they are also able to pass through intersegmental membranes (11). The *Steinernema* species, although lacking such a structure, are also able to pass through the integument favored by the high hydrostatic pressure characteristic of small nematodes, the size of the anterior part of their body (ca. 8–15 μm), and the secretion of histolytic enzymes. Thus, due to the absence of an epicuticular protective layer that would otherwise block the action of the histolytic enzymes produced by the IJs, *Steinernema feltiae* overcome the tegument barrier of the larvae of the Diptera species *Tipula paludosa* Meigen and *T. oleracea* L (12). Once in the hemolymph, the IJs release the symbiotic bacteria present in their gut: the *Steinernema* release *Xenorhabdus* spp. and the *Heterorhabditis* release *Photorhabdus* spp. The nematodes act like a small syringe to inject the bacteria. In the hemolymph, these bacteria multiply rapidly and produce a wide range of toxins and exoenzymes that kill the host, turning its tissues into a kind of soup on which the nematodes feed to reach the adult stage after four stages of development. Antimicrobial substances that are also secreted promote the development of symbiotic bacteria and nematodes (13). Additionally, the nematodes themselves make significant contributions to killing the host (14–19). When they reach the adult stage, *Steinernema* spp. mate and produce successive generations, whereas IJs of the *Heterorhabditis* spp. develop into self-fertilizing hermaphroditic females that will produce males and females in the next generation. The cycle is completed within a few days, and hundreds of thousands of new IJs will emerge from the now- destroyed host in search of new victims. Invasion of a victim by a single individual of *Heterorhabditis* is enough to produce a new generation, whereas at least two IJs are needed in the case of *Steinernema*, being gonochoric (20). When the food supply becomes scarce, development stops at the IJ stage and the cadaver is abandoned (**Figure 3**). *Steinernema* and *Heterorhabditis* spp. parasitize a wide range of insect species, and the duration of the biological cycle is influenced by both the environmental temperature and the species/strain of the nematode itself. Usually, the death of the host occurs quickly, as 48 hours is sufficient time for the bacteria to take effect.

## Symbiotic bacteria

**Figure 4:**
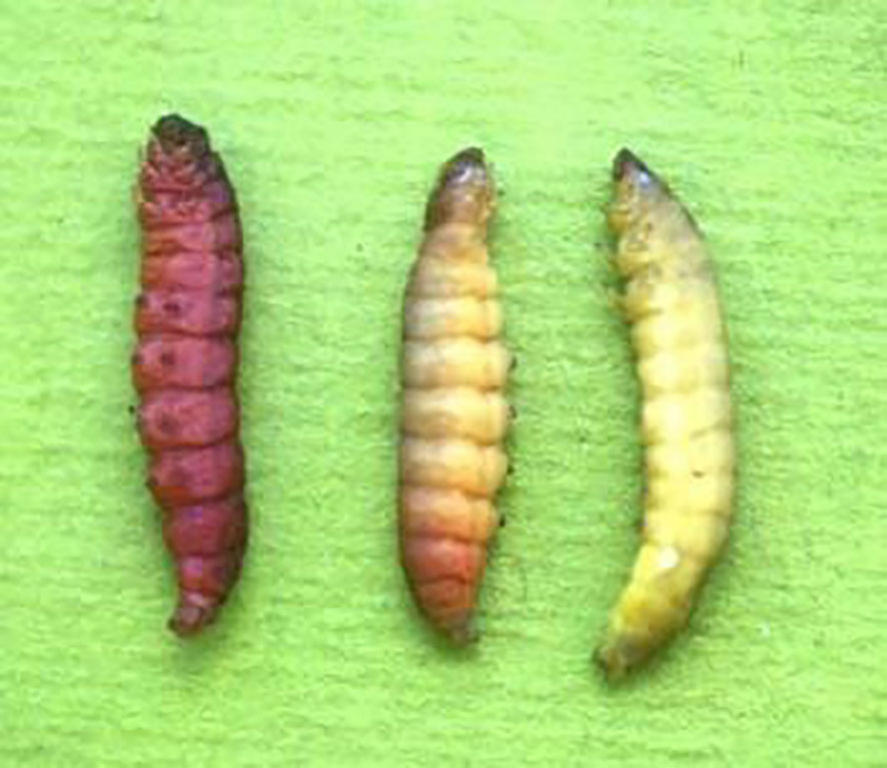
Characteristic coloring of larvae (*Galleria mellonella*) due to the action of symbiotic bacteria: *Photorhabdus* gives the larva a red or greenish color, *Xenorhabdus* a grayish or yellow-cream color.

Symbiosis with bacteria is the reason why Steinernematidae and Heterorhabditidae are more correctly called entomopathogenic nematodes and not entomoparasites. *Photorhabdus* and *Xenorhabdus*, symbionts of *Steinernema* and *Heterorhabditis*, respectively, belong to the family Enterobacteriaceae and are elongated (0.5 × 1–10 μm for the former, 0.3–2 × 2–10 μm for the latter) asporigenous Gram-negative and facultative anaerobic bacteria (21, 22). These entomopathogenic bacteria are obligate symbionts of EPN species; they biosynthesize and release secondary metabolites on artificial substrates with antibiotic activity against Gram-positive and Gram-negative bacteria and with antifungal, nematicidal, anti-ulcer, antiviral, and antitumor activity (23–25). The nematode–bacterium symbiotic relationship results in the nematode protecting the bacterium from the external environment and introducing it into the host, with the bacterium providing food for the nematode to develop. *Xenorhabdus* reside in a special bladder located just behind the basal bulb of the esophagus present in the infective stages of *Steinernema* (26), whereas *Phothorabdus* are housed in the middle part of the intestine of *Heterorhabditis* IJs (27). Although bacteria and nematodes can reproduce separately, together they have high specificity (26). However, there are some exceptions, such as *X. bovienii* (which is a symbiont not only of *S. affine* but also of *S. feltiae*, *S. ichnusae*, *S. kraussei*, *S. intermedium*, and *S. weiseri*) and *X. kozodoii* (which is associated with *S. arenarium* and *S. apuliae*) (28, 29). Among the *Photorhabdus*, *P. luminescens* and *P. luminescens laumondii* are simultaneously present in *H. bacteriophora* (30). Insects killed by the bacteria take on characteristic colorations: victims of *Xenorhabdus* become grayish or creamy yellow, whereas those with *Photorhabdus* in their tissues are deep red, or in rare cases greenish, and moderately luminescent in the dark (**Figure 4**). Both kinds of bacteria, when multiplying on artificial substrate for a long period of time, produce cells of the second type (or phase II), which have altered properties compared with cells isolated from nematodes (or phase I) and are not infectious. Phase II does not occur naturally in nematodes. Recently, in the context of this nematode–bacteria symbiosis, an infectious contribution has also been demonstrated for the bacterium *Pseudomonas protegens* (7) and, as a corollary of the description presented in this paragraph, it is important to point out the significant work of updating and deepening the field’s knowledge of EPN-associated microbiota that has been carried out by the Gaudriault lab (31).

## Attack strategies and insect reactions

The strategies used by IJs to approach their victims vary according to the species of nematode. There are two types: the ambush strategy and the cruiser strategy (32). Species that use the ambush strategy include *Steinernema carpocapsae* and *S. scapterisci*, which wait for the victim with an upright body and reach it with a jump up to 5 mm long; their action is mainly directed against arthropods that are present and active on the soil surface. *Heterorhabditis* spp., *Steinernema glaseri*, and *S. apuliae* are more mobile and actively search for prey in the soil (the cruiser strategy), while *S. riobrave*, *S. feltiae*, and *S. ichnusae* (33) use an intermediate strategy. The success of the attack is also influenced by the soil type, which may hinder or favor the movement of the nematodes in search of a victim (34, 35). One of the most important systems of insect defense against pests is encapsulation (36). However, this is ineffective against microorganisms and parasites that have developed strategies to bypass or inactivate the immune system of the host. In other cases, the behavior of the potential victim is more direct and tends to avoid penetration by the IJs. This behavior is observed in the larvae of *Popillia japonica* Newman (Coleoptera, Scarabaeidae), which dispose of the IJs of *H. bacteriophora* by wiping their body with their legs and rubbing the posterior part of the abdomen to avoid penetration via the anal opening (37).

## EPN surveys, sampling, rearing, and preservation

**Figure 5:**
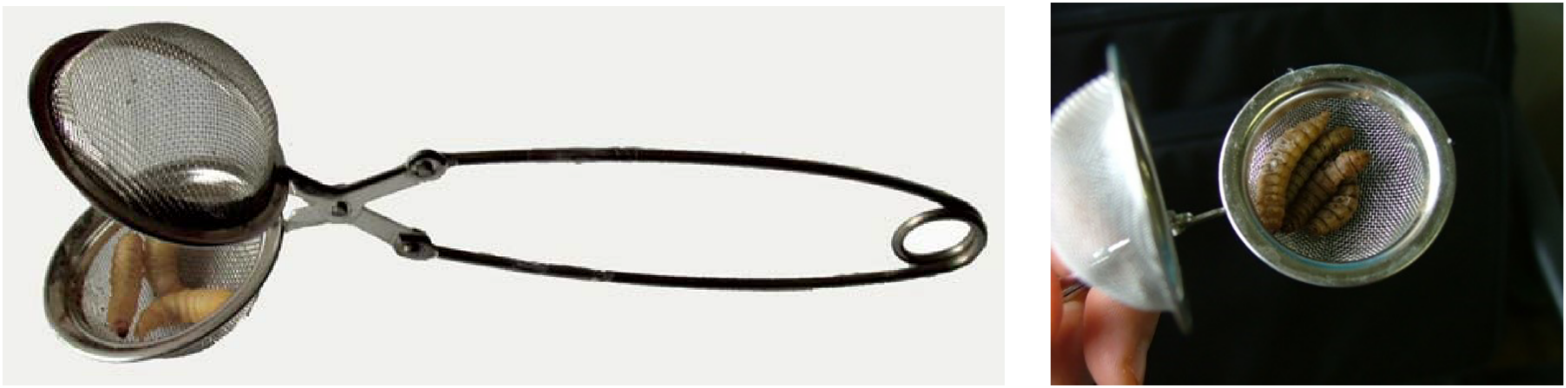
*Galleria mellonella* larvae in a stainless steel tea filter.

**Figure 6:**
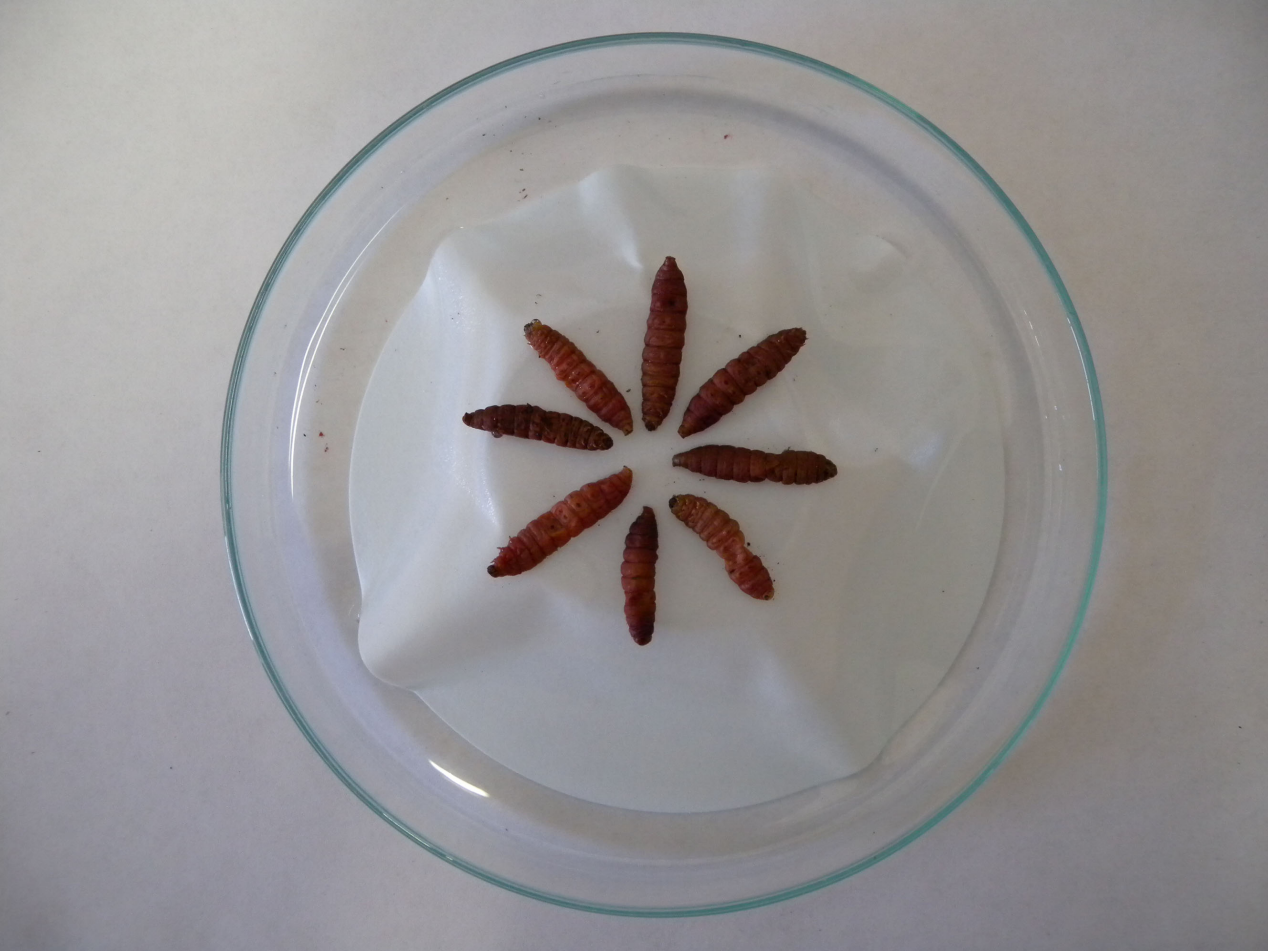
“White trap” for collecting EPNs from infested *Galleria mellonella* larvae.

The most practical method for obtaining IJs from soil is to collect representative soil samples rather than taking a single large sample or using a soil probe (38, 39). This is because taking a single sample reduces the probability of obtaining EPNs. In the laboratory, samples weighing approximately 1 kg are then placed in plastic bags, moistened with water as needed, and placed in a wire mesh cage containing between two and five larvae of the lepidopteran *Galleria mellonella* L. (Lepidoptera, Pyralidae) to attract nematodes. Other researchers prefer to use wire mesh tea filters instead of cages to prevent *Galleria* larvae from dispersing in the soil and being attacked by predators (**Figure 5**). These filters are equipped with a long stem and can also be inserted directly into the soil in the open field. For *in vivo* nematode production, last-stage larvae of *G. mellonella* are infested with suspensions of IJs in a Petri dish containing two moist filter papers at the bottom, or alternatively, a 2- to 3-mm-thick layer of well-washed and sterilized sea sand. After 3 to 5 days, the dead larvae are transferred to “White traps” (**Figure 6**) to promote the multiplication of EPNs, and the IJs are subsequently collected (40). These are then washed several times in sterile water and stored in the refrigerator. Pieces of sponge soaked in suspensions of IJs in water are used for this purpose, consisting of 500–1,000 IJs per cm^2^ of sponge in a tightly sealed plastic bag to avoid dehydration. At 5°C–10°C, the life span of the infective stages is between 1–3 months and several years, depending on the species. This type of preservation requires little skill and little energy, and the nematodes are of good quality. The disadvantage is the high cost (approximately €1 per million nematodes) and the lack of scale efficiency. In addition to refrigeration, entomopathogenic nematodes can be stored indefinitely in liquid nitrogen (41). Experiments on cryopreservation have been and are currently being conducted in Italy, with interesting results (42, 43). Bioreactors are used for industrial production (44), but currently not in Italy, where there are no production centers at present, although the country has greatly contributed to the development of technologies for the mass production of EPNs in recent decades (e.g., the Ecogen Europe biofactory, operative in Pantalla di Todi in the 1980s). In EPNs, as in all other nematodes, morphological and morphometric characters are very important for species identification. However, the high uniformity of the blueprint of EPNs, combined with the high intraspecific variability, makes identification based on morphology alone very difficult. The molecular approach involving the sequencing of specific loci, which has recently been increasingly used in the identification of animal and plant organisms in conjunction with morphological studies, has now made species identification of EPNs easier and more reliable. Under the morphological examination approach, in both families (Steinernematidae and Heterorhabditidae), the form primarily considered for species identification is the infectious juvenile third stage, i.e., the IJ.

**Figure 7:**
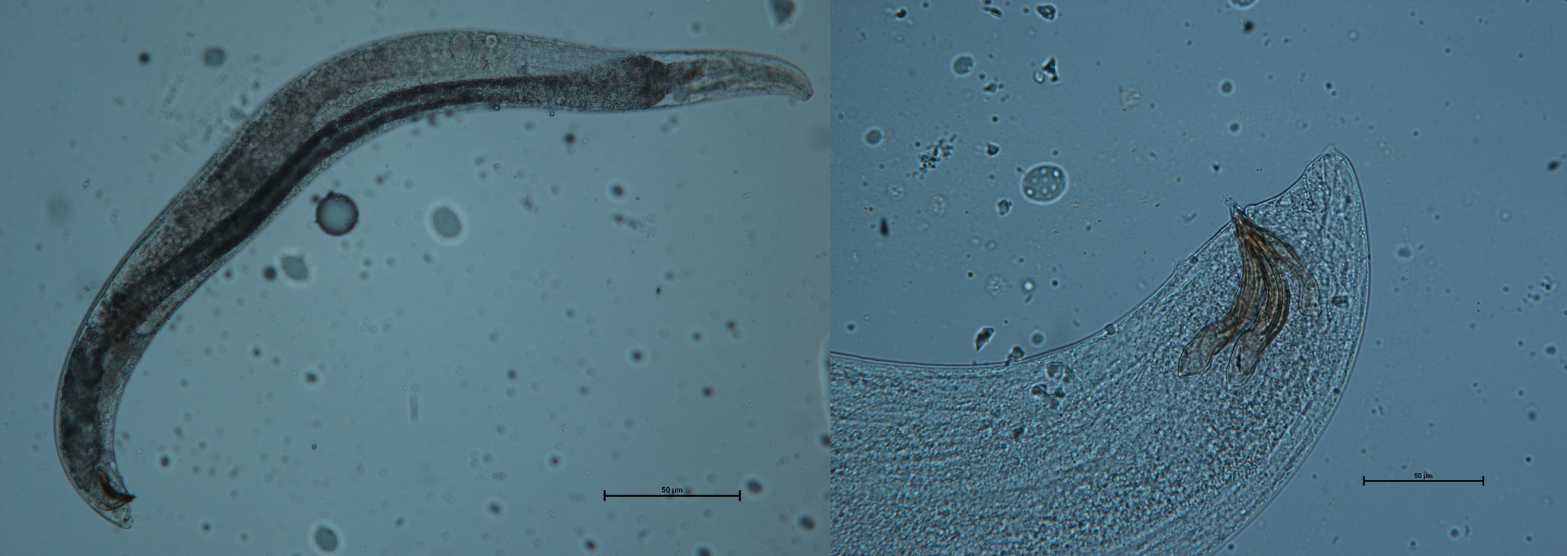
*Steinernema feltiae* It-Sf-MA12 male and particularity of the spicule.

Genus *Steinernema* Travassos, 1927 (Steinernematidae, Panagrolaimomorpha, and Strongyloidoidea) (**Figure 7**): first and second generation of amphimictic adults. Oviparous or ovoviviparous. The genus *Neosteinernema* Nguyen et Smart, 1994 (found only in America), also belongs to the same family. The genus *Steinernema* currently includes 108 species. Of the *Steinernema* species described, 15 have been found or reported in Europe and/or non-European Mediterranean regions.

**Figure 8:**
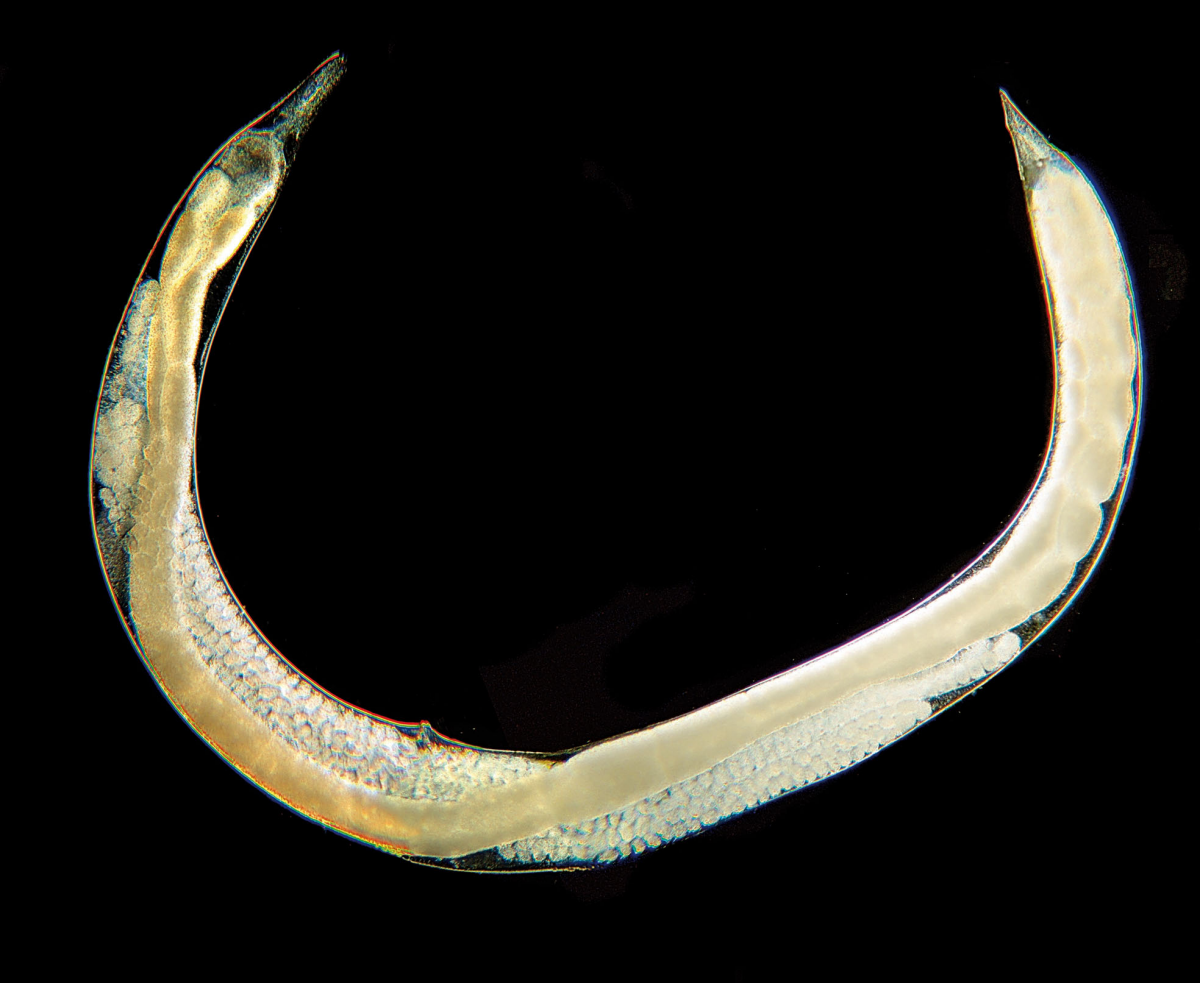
*Heterorhabditis bacteriophora* It-Hb-LU1 female.

Genus *Heterorhabditis* Poinar, 1976 (Heterorhabditidae, Rhabditomorpha, and Strongyloidea) (**Figure 8**): the first generation of adults is hermaphroditic, the second generation of adults is amphimictic. Oviparous or ovoviviparous. Fourteen species are currently classified in the genus *Heterorhabditis*, four of which have been found or reported in Europe (for details on the systematics of EPNs, see (45).

The first data on the isolation of a strain of EPNs in Italy date back to the early post-war period and concern the discovery of a small number of specimens of the beet weevil *Temnorhinus mendicus* Gyll. (Coleoptera, Curculionidae) infested with nematodes, which were described as *Neoaplectana menozii* Travassos, now a *species inquirenda* (37). However, the first samplings aiming to assess the spread of entomopathogenic nemotodes in the soil, which were carried out in the Emilia-Romagna region, date back only to 1983 (46). Subsequent research, involving almost all Italian regions, has provided and continues to provide very interesting data, including the discovery of new species. To date, more than 8,000 soil samples have been taken from different localities and biotopes. Among all sampled areas, 50% are agricultural habitats (olive groves, vineyards, orchards of various types, and vegetable and cereal fields), 35% are wooded environments (pine forests, oak forests, and chestnut forests), 10% are coastal areas, and the remaining 5% are pastures, fallow land, salt marshes, and lakeshores. To date, a total of more than 150 EPN strains have been isolated in Italy: 48 isolates of *Heterorhabditis bacteriophora*, 1 of *H. downesi*, 58 of *Steinernema feltiae*, 11 of *S. affine*, 4 of *S. kraussei*, 8 of *S. apuliae*, 4 of *S. ichnusae*, 12 of *S. carpocapsae*, 1 of *S. vulcanicum*, 4 of *S. arenarium*, and 1 of *Oscheius onirici* (the genus *Oscheius* includes several species of nematodes, of which only a few share similarities in parasitic action toward insects with EPNs). Some recently isolated strains from Lombardy have also been identified (47, 48). *Steinernema kraussei* has been isolated only in the soils of the chestnut groves of Etna, Sicily (47); *S. apuliae*, *S. ichnusae*, *S. vulcanicum*, and *O. onirici* are four new species presently recorded only in Italy, with the first isolated on the Apulian coast, the second in Sardinia and Campania, the third on the slopes of Etna in Sicily, and the fourth in Tuscany (49). A survey of the presence and characterization of entomopathogenic nematodes in Italy showed that EPNs were found in all habitats studied, indicating a wide distribution of species in different ecosystems. Steinernematidae occurred more frequently than Heterorhabditidae, with *S. feltiae* and *H. bacteriophora* being the most widespread species. *Steinernema feltiae* was isolated in most habitats, with a preference for sandy soils. *H. bacteriophora* is also a fairly common species, also found in volcanic soils (on the volcanic island of Pantelleria) but never in deciduous forests, showing a preference for sandy soils (58% of strains). Aside from the two dominant species, *S. feltiae* and *H. bacteriophora*, EPNs tended to correlate with a specific habitat: *S. kraussei* and *S. affine*, for example, were found in forests at fairly high altitudes; *S. affine* was isolated from different soil types, but almost exclusively in deciduous forests; and *S. kraussei* was isolated in chestnut forests with sandy soils on Mount Etna in Sicily (46). Concerning habitat preferences for other species, *S. apuliae* was isolated from different habitats, but always near coastal areas, showing a clear preference for sandy soils, while *S. carpocapsae* was isolated in the northern part of Apulia and in Tuscany, Emilia-Romagna, Lombardy, and Veneto, mostly in fallow soils (47). Sampling in Sicily revealed the first Italian strains of *H. megidis* and *H. downesi*, indicating that all Heterorhabditidae species reported in Europe are present in Sicily. Soil characteristics also influence the presence of EPNs, and our survey showed a clear correlation between EPN presence and soil texture, with EPNs exhibiting a preference for sandy and medium-textured soils. No strains were isolated from clay soils. This is most likely because sandy and medium-textured soils promote EPN mobility and survival, whereas clay-rich soils restrict nematode movement. The importance of two natural habitats, pine forests and oak forests, is suggested by this study of EPN distribution among habitats. These two forest environments have the highest concentrations of isolated EPN strains, with only three of the seven species found in the forests also being found in other habitats. The data on the biodiversity of EPNs collected thus far do not exhaustively cover all geographic areas and habitats in Italy, but they still contribute significantly to our understanding of the presence and geographic distribution of EPNs in relation to the wide variety of habitats found in Italy (50).

## Identification of EPN strains via DNA analysis

Molecular approaches show particular accuracy where morphological characterizations at the species level are not able to discriminate between closely related species. Molecular techniques based on PCR allow the analysis of informative DNA markers and genes from individual nematodes. A molecular marker is any DNA sequence that is widespread throughout the genome showing polymorphism (51) that can be detected using molecular techniques. Markers must also be stable, enabling their identification in each developmental stage of nematodes (52). The target regions used for EPN identification are nuclear, ribosomal, and mitochondrial DNA. The cistron of ribosomal DNA contains three highly conserved genes, *18S rRNA*, *5.8S*, and *28S*, and highly variable regions such as the internal transcribed spacer (ITS; ITS1 and ITS2), IGS (intergenic spacer), and ETS (external transcribed spacer) regions. The ITS and expansion domains of the *28S* gene are the most variable regions of rDNA and, along with partial mitochondrial genes, such as NADH dehydrogenase subunit 4 (*nd*4) or cytochrome oxidase (COI), and protein-coding genes, such as *cmd*-1, *unc*-87, *hsp*70 (53, 54), and *hsp*90 (55), are very useful markers for species differentiation. Furthermore, they allow the detection of misidentifications in order to establish phylogenetic relationships within and among EPNs (56–64). Recent studies by Spiridonov and Subbotin (65) and by Dhakal et al. (64) have confirmed that the ITS, the *28S rRNA* gene, the COI, nuclear *cmd*-1, and *unc*-87 sequences are useful markers for EPN identification, confirming the occurrence of three groups within the *Heterorhabditis* genus, namely, “Indica”, “Bacteriophora”, and “Megidis”. Sequence analyses have also enabled the detection of misidentifications in GenBank and correction of their taxonomic status within the *Heterorhabditis* species. Furthermore, pairwise analyses permit the estimation of intraspecific and interspecific sequence variability within most of the *Heterorhabditis* and *Steinernema* species and strains (64). For Italian EPNs (Heterorhabditis, Steinernema, and Oscheius strains), the markers mainly used are ITS regions, the most conserved region of the D2D3 domain (LSU) for ribosomal DNA, the cytochrome oxidase I (COI) locus, and a partial portion of hsp90 (55, 66). Fanelli et al. (55) used degenerate primers to obtain partial *hsp*90 gene sequences from several entomopathogenic nematodes, including *H. bacteriophora* and *O. myriophilus*, and demonstrated that phylogenetic trees based on *hsp*90 sequences showed equal resolution, and in most cases were congruent with those inferred from ribosomal markers.

Comparison of the ITS sequences among *Steinernema* species has also showed higher variability than either the D2-D3 expansion domains of the *28SrRNA* gene or the partial *18S rRNA* gene (61). Analysis of partial mitochondrial COI sequences among *Steinernema* species has revealed that fewer clades could be resolved by this method than with ITS (61), despite the plurality of informative sites available for COI sequences.

**Figure 9:**
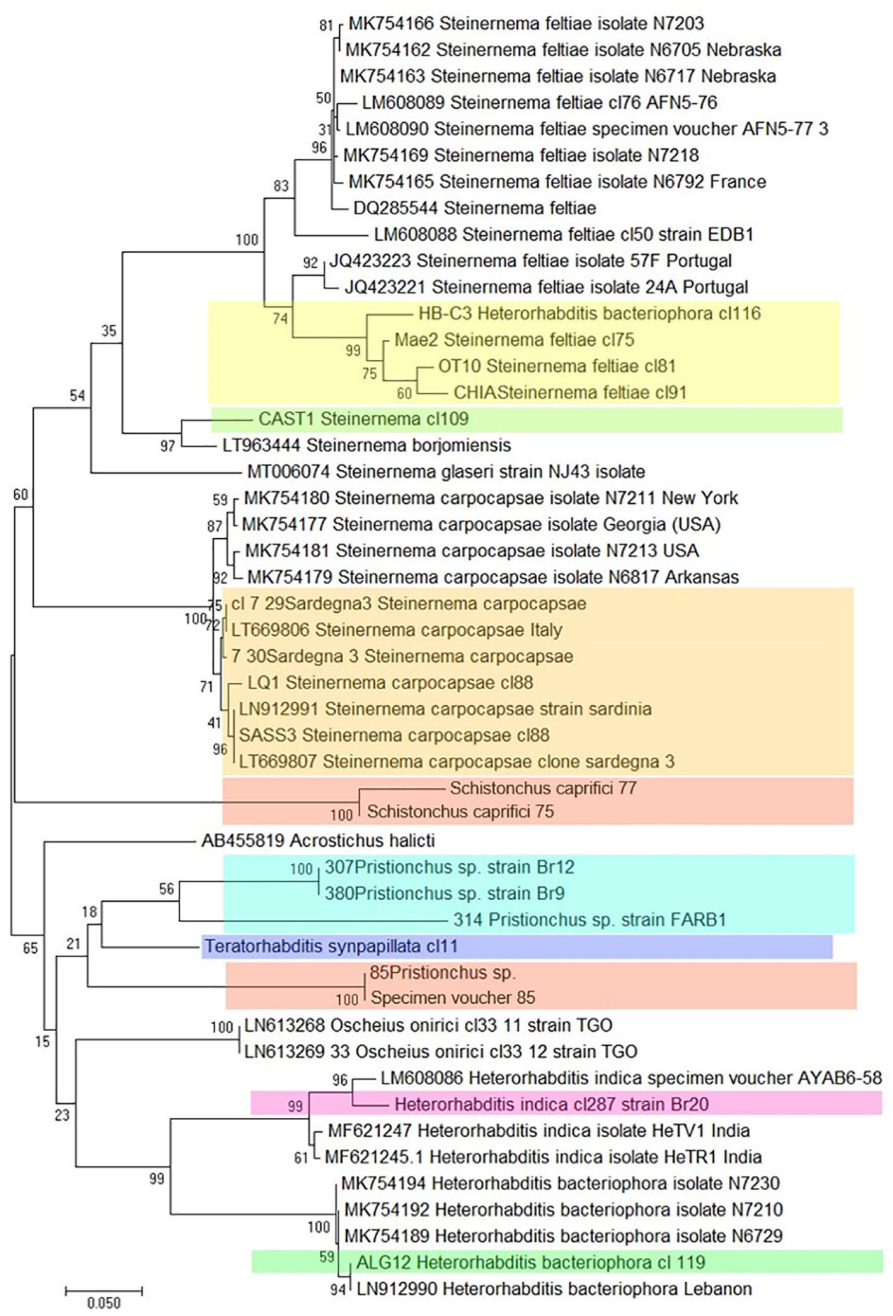
Phylogenetic relationships of COI among different Italian strains of entomopathogenic nematodes, based on sequencing of several clones of each strain. (Colored boxes indicate the Italian strains).

Massive sequencing techniques have produced a large number of ITS and COI sequences for EPNs, leading to the development of the “DNA barcoding” protocol for the rapid identification of nematodes. This is a molecular method dedicated to the identification of biological identity that involves testing the variability of a relevant marker (67, 68). This approach exploits conserved regions of these genes, comparing all sequences present in the database in order to design universal primers that amplify short DNA fragments to be used as a barcode, and identifying even the cryptic nematode species (which have the same morphology but which are genetically different) and larval stages present in a soil sample, even in the absence of morphological identification (2, 67). Molecular barcodes can group an unknown EPN specimen phylogenetically into its correct position compared to known reference sequences (**Figure 9**).

### PCR-RFLP, RAPD, SRAP

Sequence differences in PCR products from different EPN spp. or strains can be analyzed by digestion with restriction enzymes. Six restriction enzymes are sufficient to differentiate almost all nematode species. Restriction fragment length polymorphism (RFLP) profiles of the ITS region are accurate and reproducible, allowing characterization of *Steinernema* and *Heterorhabditis* species (43, 69). ITS-RFLP analyses can also reveal the presence of different strains within EPN species, thus indicating genetic variability due to geographical origin or insect host (70, 71).

The random amplification of polymorphic DNA (RAPD) technique enables measurement of genetic diversity in EPNs, even if no prior sequence information is available. This technique involves the amplification of gDNA fragments using a single primer of 10 nucleotides with an arbitrary (random) sequence that can bind to complementary sequences present in the DNA. The profiles of the fragments generated are species-specific and can be compared with those of other EPN species (72). The presence or absence of a fragment in a sample represents a diagnostic marker, allowing researchers to discriminate among and within *Steinernema* spp. and *Heterorhabditis* spp (73–75).

Several tools, such as amplified fragment length polymorphism (AFLP), simple sequence repeats (SSR), inter-simple sequence repeats (ISSR), single-strand conformation polymorphism (SSCP), and sequence-related amplified polymorphism (SRAP), are also used to characterize genetic diversity in EPN species (76–79). SRAP has been successfully used to evaluate genetic diversity in EPN species, and a study by Youssef et al. (80) revealed that 12 different SRAP primer pairs can be used to differentiate among seven *Steinernema* species, with this method being more accurate than RAPD.

### Real-time PCR

The real-time PCR (qPCR) technique enables monitoring of the amplification in real time and quantification of the number of copies of a specific target region. Copy number quantification of the target region is carried out during the exponential phase of PCR, producing much more accurate results than the traditional PCR “end point”. Quantification of the product is achieved by adding fluorescent compounds that are incorporated into each copy of the amplified product in each cycle, and fluorescence is proportional to the quantity of the amplified product. At present, several real-time qPCR probes are available to characterize EPN assemblages in the field (81–84). Species-specific primers and probes have been designed based on the ITS rDNA sequences of several *Steinernema* and *Heterorhabditis* species, and are available to the scientific community (82, 84, 85).

Real-time PCR (qPCR) enables not only discrimination among different species but also exploration of the relationships and the functions of different organisms within ecosystems (83). Thus, qPCR and species-specific primer–probe combinations allow the simultaneous detection, identification, and quantification of EPNs, as well as their antagonists and competitors, in soil samples (83, 84).

Sequencing technologies, such as high-throughput sequencing (HTS) and next-generation sequencing (NGS), are evolving rapidly, allowing accurate EPN identification and analysis of soil nematode communities (86). Furthermore, metagenomic analysis can explore EPN biogeography and the factors that modulate the presence and abundance of these organisms in soil samples (87).

### Loop-mediated isothermal amplification

Recently, loop-mediated isothermal amplification (LAMP) assay has been employed for rapid detection of *Heterorhabditis* spp. and *Steinernema* spp. from total soil DNA (88).

LAMP was developed in 2000 (87, 88) and has been used for the identification of animal and plant pathogens, including plant parasitic nematodes. It combines the simplicity and rapidity of reaction setup with ready data interpretation. The advantage of the LAMP method is that DNA amplification reactions take place at a constant temperature. LAMP is based on the isothermal amplification of nucleic acids by thermostable polymerase, with “strand displacement” activity isolated from *Bacillus stearothermophilus* (BSt polymerase) and on six specific primers which recognize eight regions on the selected target DNA (F3c, F2c, and F1c in the 3′ direction and B1, B2, and B3 in the 5′ direction). LAMP is inexpensive, fast, and reproducible, and can be conducted even in poorly equipped laboratories (89–91).

LAMP primers for *Heterorhabditis* and *Steinernema* have been designed by aligning sequences of the ITS region of rDNA for *Heterorhabditis* spp. and the 18S rDNA sequences for *Steinernema* spp. that were retrieved from GenBank. Five separate sets of LAMP primers are available for each EPN genus (88). The LAMP technique reduces the time and labor costs associated with the insect-baiting technique and provides information that can be used in the development of detection kits for the diagnosis of EPNs in the field without the need for trained and experienced personnel.

## Biological control strategies using EPNs

**Figure 10:**
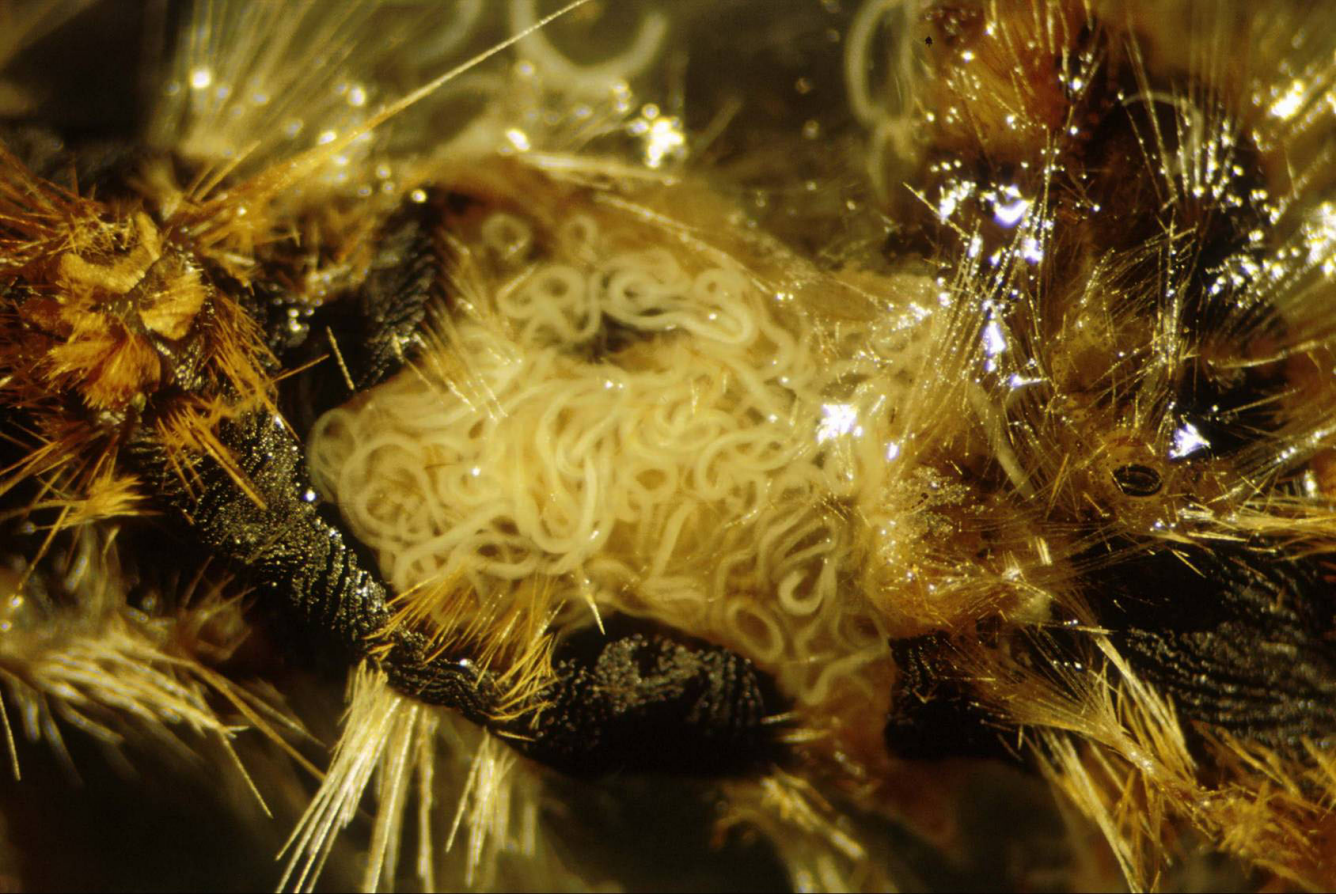
Interior of *Thaumetopoea pityocampa* larva invaded by *Steinernem*a *feltiae*, Italian strain.

**Figure 11:**
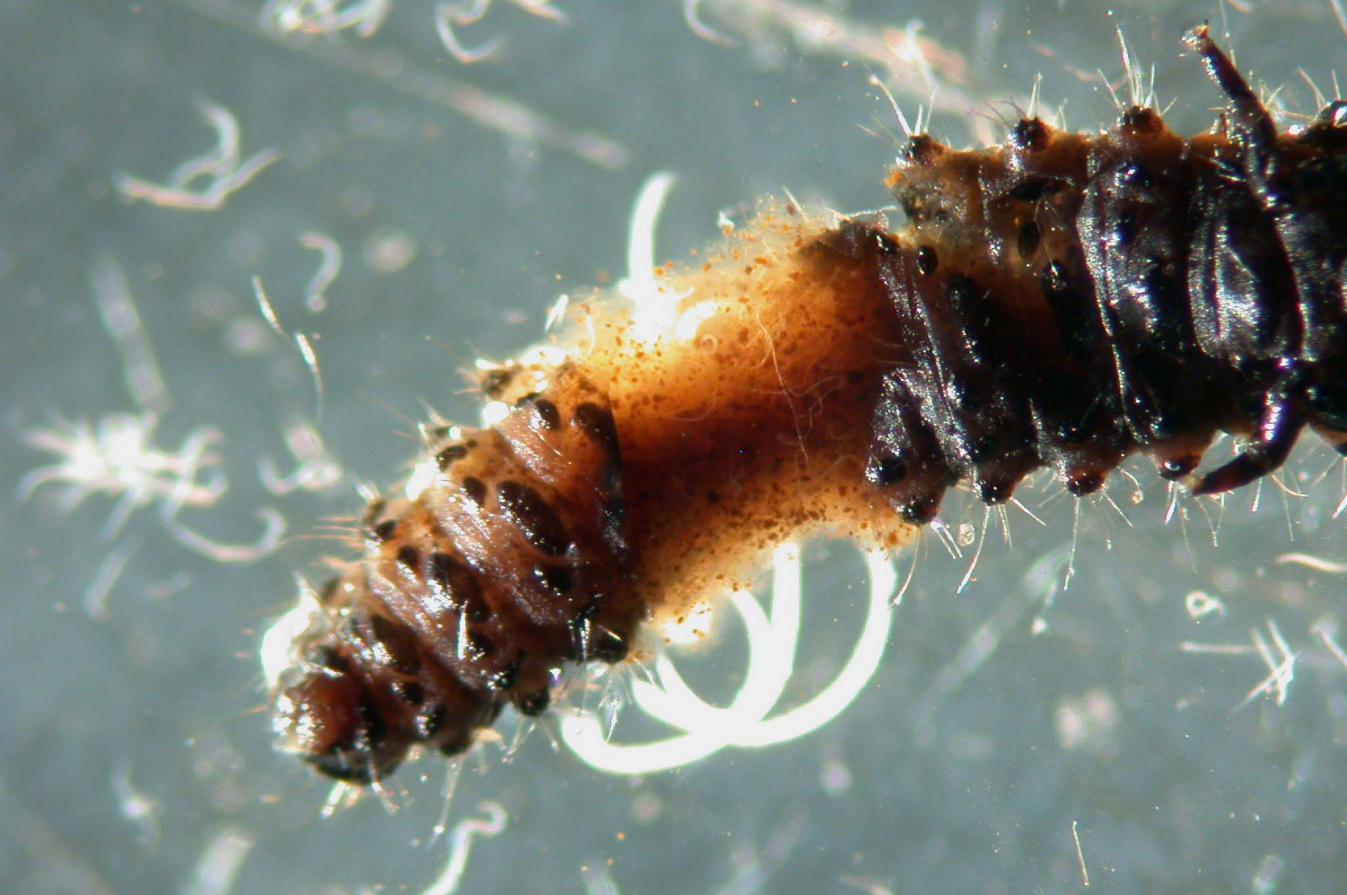
*Xanthogaleruca luteola* larva infested by *Steinernema feltiae*, Italian strain.

**Figure 12:**
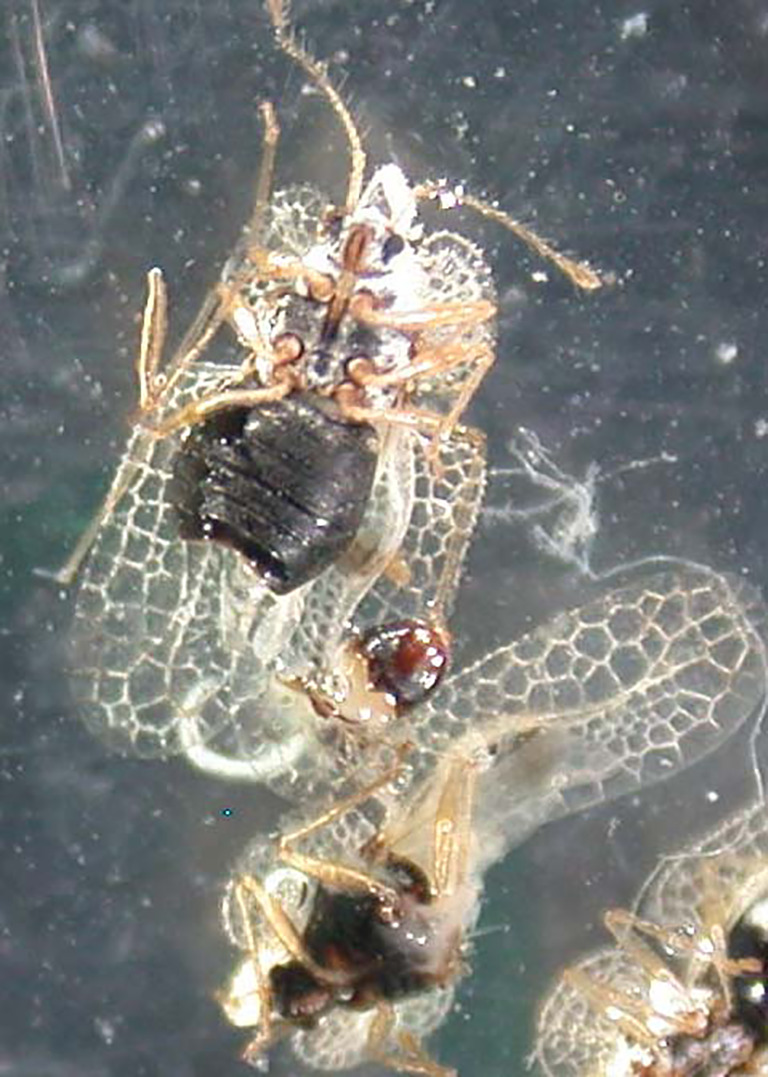
*Corythucha ciliata* adults infested by *Steinernema feltiae*, Italian strain.

**Figure 13:**
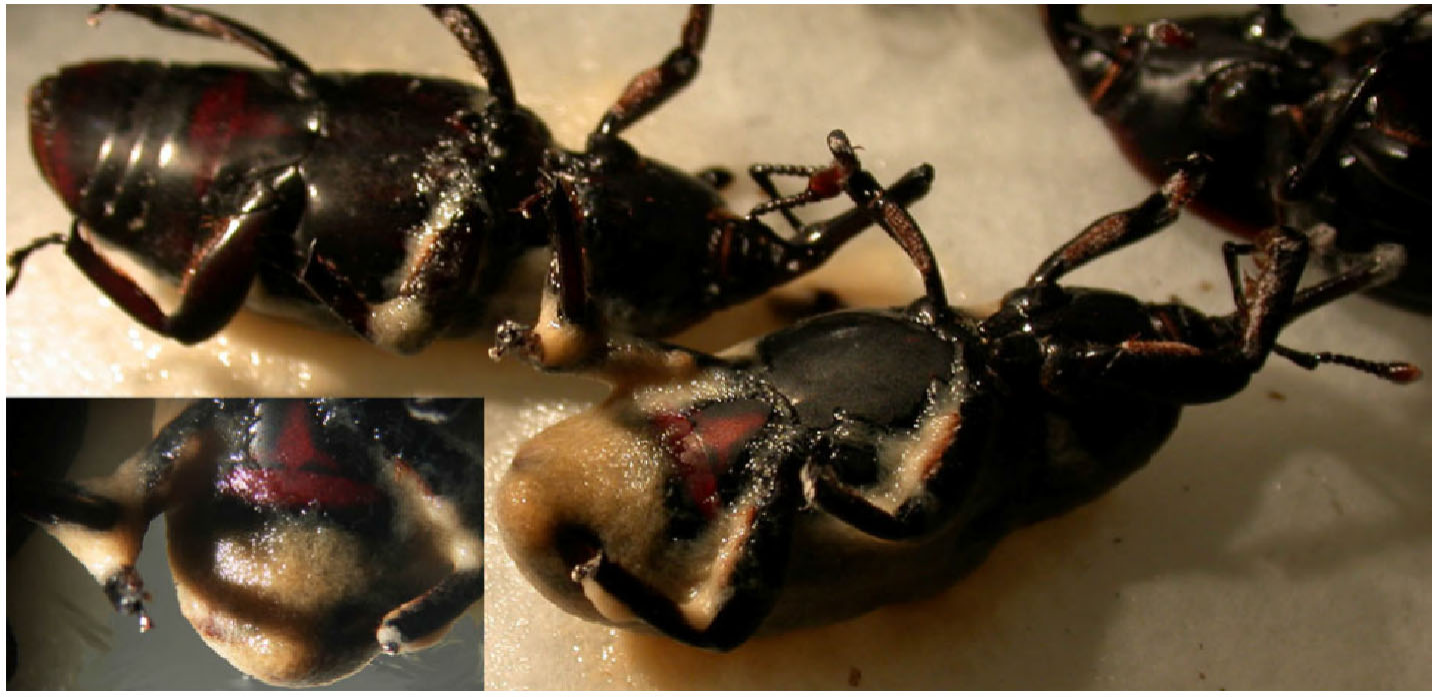
*Rhynchophorus ferrugineus* adults infested by *Heterorhabditis bacteriophora*, Italian strain.

Commercial products containing *Heterorhabditis bacteriophora*, *H. megidis*, *Steinernema feltiae*, and *S. carpocapsae* have been successfully used to control various species of Heteroptera, Lepidoptera, Coleoptera, Diptera, and Hymenoptera (50). In Italy, EPN formulations have been effectively tested and used to control various species, including but not limited to the following: the scolytid *Tomicus piniperda* (92) and the lepidopteran *Thaumetopoea pityocampa* on pines (93) (**Figure 10**); the lepidopterans *Pammene fasciana*, *Cydia splendana*, and *C. fagiglandana* and the coleopterans *Curculio elephas* and *C. glandium* on chestnut trees (94, 95); the lepidopteran *Cydia pomonella* and the hymenoptera *Hoplocampa brevis* on pear trees (95, 96); the curculionid *Rhytidoderes plicatus* on cabbage roots (97); the chrysomelid *Xanthogaleruca luteola* on elm trees (98) (**Figure 11**); the tingid *Corythucha ciliata* on sycamores (99) (**Figure 12**); and the curculionid *Otiorhynchus sulcatus* on ornamental plants (100) and on strawberries (101, 102). In the Mediterranean region, because of the spread of exotic palm weevils, promising experiments to contain the spread of the weevil *Rynchophorus ferrugineus* with entomopathogenic EPN nematodes have been carried out in several countries, such as Egypt, Spain, and Italy (**Figure 13**). Other applications of beneficial nematodes have been effective in controlling sciarid dipteran in ornamental nurseries and mushroom farms, the weevil curculionid *Curculio nucum* on hazels (103), the thrips *Frankliniella occidentalis* (104), the lepidopteran *Tuta absoluta* on tomato, the chrysomelid *Diabrotica virgifera* on corn (105), and slugs and snails in horticulture, with the latter case involving the use of the specific nematode *Phasmarhabditis hermaphrodita* (49). Entomopathogenic nematodes have also been found to be effective against xylophagous insects residing in cryptic habitats, such as *Capnodis tenebrionis*, *Arhopalus syriacus*, *Cossus cossus*, and *Parahypopta caestrum* (106). Recently, these biological control agents have found broad application in integrated control programs of the Scarab beetle *Popillia japonica* in large cultivated and non-cultivated areas of Lombardy and Piedmont (105, 106), and of the cossid *Parahypopta caestrum* in the asparagus fields of Apulia (107).

### Examples of biological control strategies with EPNs

The larvae of Coleoptera Curculionidae of the genus *Otiorhynchus* represent the classic example of a target pest of EPNs, to the extent that this type of biological control is currently widely tested and used around the world. The most suitable nematodes against *Otiorhynchus* larvae belong to the genus *Heterorhabditis*, although satisfactory results have been achieved with formulations based on *S. feltiae* and, to a lesser extent, on *S. carpocapsae* (108, 109). Formulas based on *H. bacteriophora* are marketed in Italy in packages containing 50 to 250 million IJs. The doses used correspond to 200,000 to 400,000 nematodes per m^2^, or 25,000 to 40,000 nematodes per plant (50). The end of summer is the best time for application, so as to target the early larval stages of the insects, which are more sensitive and are at the beginning of their phytophagous action, under optimum soil temperatures of 18°C –22°C (110). Spring treatments on larger overwintering larvae and newly formed pupae are equally effective if soil temperatures are above 15°C and a dose of 500,000 nematodes per m^2^ is used (50). The crops targeted for this type of application are typically ornamental plants in nurseries and strawberry crops in open field and protected cultivations. Another example EPN application, whose use has expanded in recent years to cover a surface area of 800 ha in Emilia-Romagna and approximately 1,500 ha throughout northern Italy, is that of the autumn treatments used against overwintering larvae of *Cydia pomonella* on pear and apple (50). The nematodes used belong to the species *S. feltiae* and *S. carpocapsae*, which are applied to the trunks and lower branches of the trees, where the larvae overwinter in bark crevices, protected in a light cocoon that the nematodes can perforate (49). The application dose corresponds to 1.5 × 10^9^ IJ diluted in 15 hL of water per ha, distributed by the company atomizer, for which it is necessary to close the highest nozzles and remove the filters (49). Thorough moistening of the plants and soil before and after the application of entomopathogenic nematodes and constant moistening of the substrate promote good efficacy of the biological treatment and ensure parasitization of the insect; if water availability is limited, it is advisable to inject the EPN suspension into the soil. Xylophagous insects, such as Lepidoptera Cossidae (*Cossus* L. and *Zeuzera pyrina* L.), Coleoptera Cerambycidae (*Saperda carcharias* L.), and Buprestidae (*Capnodis tenebrionis* L.) can also be effectively controlled with injections of IJ suspensions into the penetration holes (also with the addition of chitosan) or by the obstruction of these openings with pieces of sponge soaked in nematodes. EPNs are typically applied to culture systems and substrates that are regularly treated with other chemicals, including natural soil improvers and fertilizers. *Heterorhabditis bacteriophora*, *S. carpocapsae*, and *S. feltiae* are in most cases compatible with plant protection products, but can interact with such substances, in some cases even producing antagonistic or synergistic effects (111).

## Regulation of the use of entomopathogenic nematodes

EPNs are ubiquitous organisms that are not dangerous to higher animals or humans and do not have any side effects for plants. Before being used, entomopathogenic organisms should, in general, be subject to registration screening, with the exception of ENPs, which are generally exempted due to their pluricellular structure and recognized specificity to insects. At the European level, Regulation (EC) No. 1107/2009 of the European Parliament and of the Council of 21 October 2009, which repealed Commission Directive 91/414 concerning the placing on the market of plant protection products, applies to active substances, including micro-organisms, safeners, synergists, co-formulants, and adjuvants, that have a general or specific action against harmful organisms. This regulation provides for the registration of three “categories” of formulations: biocides, microorganisms, and viruses. Nematodes and macro-organisms (insects and auxiliary mites) are not mentioned, pursuant to the principle of forgoing the registration process for products with low environmental impact, based on the unequivocal interpretation of the countries of the European Union. However, unlike those countries that currently do not require any registration (such as Denmark, Finland, France, Greece, Germany, Italy, Portugal, and Spain), others do require some form of registration: Austria requires the same form of registration as that used for chemicals; Belgium and the Netherlands require registration only for new formulations; Poland, the Czech Republic, and Hungary provide for a preliminary field trial; Ireland, Switzerland, Norway, and Sweden require the registration of all biological control agents; and in the UK, no registration is required for indigenous ENPs, but the introduction of non-native strains in the wild in the country is controlled by the Wildlife and Countryside Act (112). In Japan, registration is required for ENPs as well as for chemicals, whereas in other non-European countries (such as Australia, Canada, and the United States), no registration is needed, provided that the ENPs are indigenous. In New Zealand, in contrast, native species must also be registered and are protected. All these states have also enacted specific legislation to regulate the import and release of non-native species. The REBECA Action (Regulation of Biological Control Agents, 113), funded by the European Union for the correct use and marketing of EPNs, has made several recommendations on the use of entomopathogenic nematodes, such as knowledge of the exact identity (specific identification) of the ENPs and accurate identification of the symbiotic bacterium of *Heterorhabditis indica*, to exclude the presence of *Photorhabdus asymbiotica*, which is harmful to humans. At the same time, it has also been pointed out that no precautions are necessary if one uses native EPNs and is provided with comprehensive information on the “environmental risk assessment” (ERA) criteria used for insects and auxiliary mites. Thus, molecular biology and phylogenetic reconstruction play an important role in elucidating the systematics, cospeciation, and coadaptation of entomopathogenic nematodes and their symbiotic bacteria when new EPNs are used as biological control agents (114–116).

## Author contributions

All authors listed have made a substantial, direct, and intellectual contribution to the work and approved it for publication.
