# Supplementary figures and images for: Entomopathogenic nematodes and their symbiotic bacteria: from genes to field uses
Source: Front Insect Sci. 2023 Aug 29;3:1195254. doi: 10.3389/finsc.2023.1195254 (PMC10926393; doi:10.3389/finsc.2023.1195254)

## Slide 1
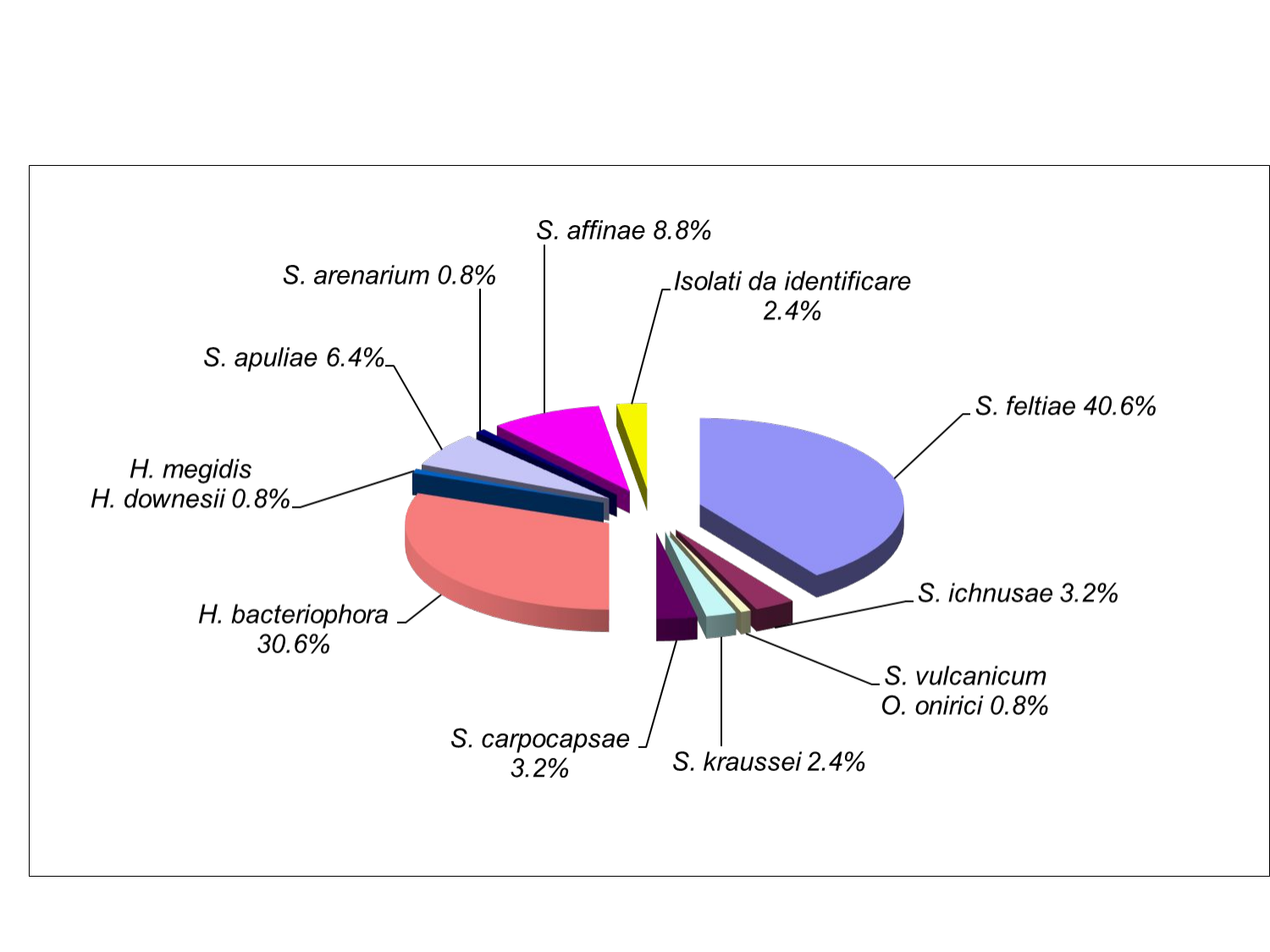

Supplement: Supplementary file 1 [file Presentation_1.ppt]
